# The Reduced Level of Inorganic Polyphosphate Mobilizes Antioxidant and Manganese-Resistance Systems in *Saccharomyces cerevisiae*

**DOI:** 10.3390/cells8050461

**Published:** 2019-05-15

**Authors:** Ludmila Trilisenko, Anton Zvonarev, Airat Valiakhmetov, Alexey A. Penin, Irina A. Eliseeva, Vladimir Ostroumov, Ivan V. Kulakovskiy, Tatiana Kulakovskaya

**Affiliations:** 1 Skryabin Institute of Biochemistry and Physiology of Microorganisms, FRC Pushchino Center for Biological Research of the Russian Academy of Sciences, pr. Nauki 5, Pushchino 142290, Russia; tl@lamalab.org (L.T.); zvonarevibpm@gmail.com (A.Z.); airatv@ibpm.pushchino.ru (A.V.); 2 Institute for Information Transmission Problems, Russian Academy of Sciences, Bolshoy Karetny per. 19 bld .1, Moscow 127051, Russia; alekseypenin@de.bio.msu.ru; 3 Institute of Protein Research, Russian Academy of Sciences, Institutskaya 4, Pushchino 142290, Russia; yeliseeva@vega.protres.ru; 4 Institute of Physicochemical and Biological Problems of Soil Science, FRC Pushchino Center for Biological Research of the Russian Academy of Sciences, pr. Nauki 2, Pushchino 142290, Russia; v.ostroumov@rambler.ru; 5 Vavilov Institute of General Genetics, Russian Academy of Sciences, Gubkina 3, Moscow GSP-1 119991, Russia; 6 Engelhardt Institute of Molecular Biology, Russian Academy of Sciences, Vavilova 32, Moscow GSP-1 119991, Russia; 7 Institute of Mathematical Problems of Biology RAS—the Branch of Keldysh Institute of Applied Mathematics of Russian Academy of Sciences, Vitkevicha 1, Pushchino 142290, Russia

**Keywords:** polyphosphate, *PPN1*, *PHM7*, *PHO84*, manganese adaptation, oxidative stress, *Saccharomyces cerevisiae*

## Abstract

Inorganic polyphosphate (polyP) is crucial for adaptive reactions and stress response in microorganisms. A convenient model to study the role of polyP in yeast is the *Saccharomyces cerevisiae* strain CRN/PPN1 that overexpresses polyphosphatase Ppn1 with stably decreased polyphosphate level. In this study, we combined the whole-transcriptome sequencing, fluorescence microscopy, and polyP quantification to characterize the CRN/PPN1 response to manganese and oxidative stresses. CRN/PPN1 exhibits enhanced resistance to manganese and peroxide due to its pre-adaptive state observed in normal conditions. The pre-adaptive state is characterized by up-regulated genes involved in response to an external stimulus, plasma membrane organization, and oxidation/reduction. The transcriptome-wide data allowed the identification of particular genes crucial for overcoming the manganese excess. The key gene responsible for manganese resistance is *PHO84* encoding a low-affinity manganese transporter: Strong *PHO84* down-regulation in CRN/PPN1 increases manganese resistance by reduced manganese uptake. On the contrary, *PHM7*, the top up-regulated gene in CRN/PPN1, is also strongly up-regulated in the manganese-adapted parent strain. Phm7 is an unannotated protein, but manganese adaptation is significantly impaired in Δ*phm7*, thus suggesting its essential function in manganese or phosphate transport.

## 1. Introduction

Inorganic polyphosphate (polyP) is a linear anionic polymer containing from several to hundreds of orthophosphate residues linked by energy-rich phosphoanhydride bonds. PolyP participates in the regulation of many cellular events [1,2]. In mammals, polyP is involved in signal transduction cascades [3,4,5,6,7,8], serves as a phosphorus reserve for apatite formation in bone growth and development [9,10,11,12], and plays a role in blood coagulation [13,14] and inflammation [15,16,17]. In bacteria, polyP and its associated enzymes are involved in stress response and virulence, in addition to the function of phosphate and energy reserve [2,18,19,20,21]. Also, it is involved in cell motility, biofilm formation, and controls the level of the stringent response factor guanosine 5’-diphosphate 3’-diphosphate (ppGpp) [2,22], although it has been shown that ppGpp is not essential for polyP synthesis in *Escherichia coli* [23]. The role of polyP in oxidative stress response is associated with its protein-protective chaperone function [24,25], and the metal ion-complexing function [26]. In yeast, the polyP activity as a phosphate reserve is well studied [27], and the enzymes of polyP metabolism are well characterized [28,29,30]. However, the role of polyP in oxidative and toxic metal stresses in yeast remains notably less explored.

Knockout mutants lacking the genes encoding polyP-metabolizing enzymes serve as models for studying the role of polyP. For bacteria, the commonly used models are the *ppk1* or *ppk2*-null strains lacking polyphosphate kinases and having a significantly decreased polyP level [2,31,32]. They provide the possibility of direct studying of the effect of a decreased polyP level on bacterial cells, including stress response.

In yeast, it is not trivial to limit polyP synthesis without non-intended disturbance in stress response due to the following. The synthesis of the major part of polyP pool is provided by the vacuolar transporter chaperone (VTC) complex of the vacuolar membrane [28,33]. The reduced polyP accumulation was observed in mutants without *VTC4* [34] and *VTC5* [33], and in knockout strains lacking subunits of vacuolar V-ATPase [35]. All such mutants either lack some components of the VTC complex, being deficient in V-ATPase assembly and stability [36], or lack V-ATPase per se. The V-ATPase itself plays a key role in the response to oxidative and heavy metal stress [37,38,39], thus its dysfunction hides possible polyP functions in stress response.

This is why we had to design strains with enhanced expression of polyP hydrolyzing enzymes. The *S. cerevisiae* strain overproducing the Ppn1 polyphosphatase is known to have a decreased polyP level [40], and can serve as a model for studying the role of polyP in stress response in yeast.

Given the role of polyP in metal ion resistance in microorganisms [41], we expected that the Ppn1 polyphosphatase overproducing strain (CRN/PPN1) would be more sensitive to manganese excess than the control strain (CRN). Instead, CRN/PPN1 had enhanced resistance to manganese. This is explained by the pre-adaptive state of CRN/PPN1 in the normal medium, revealed by the high-throughput transcriptome profiling. Furthermore, the pre-adaptive state was characterized by up-regulation of the oxidative stress response genes, which provided enhanced resistance to oxidative stress.

## 2. Materials and Methods

### 2.1. Yeast Strains and Growth Conditions

In this study we used the following *S. cerevisiae* strains: CRN (*MATa ade2 his3 ura3 ppn1Δ::CgTRP1*), CRN/PPN1 (CRN transformed with the pMB1/PPN1 Sc vector), and CRN/PPX1 (CRN transformed with the pMB1/PPX1 Sc vector). The source of the strains and the polyphosphatase activities in the cell-free extracts are given in Table S1.

The strains were maintained in the selective agarized medium [40]. The BY4743 (*MATa/α his3Δ1/his3Δ1 leu2Δ0/leu2Δ0 LYS2/lys2Δ0 met15Δ0/MET15 ura3Δ0/ura3Δ0*), BY4743-derived Δ*phm7*, and BY4743-derived Δ*vmr1* strains were obtained from the Dharmacon collection. For the experiments, the cells were cultivated in YPD containing 2% glucose, 2% peptone (Peptone from meat enzymatic digest, Fluka, Sigma-Aldrich, St. Louis, MO, USA), 1% yeast extract (CONDA/Pronadisa, Madrid, Spain), and 14 mM KH_2_PO_4_ at 29 °C and 145 rpm. For studying the resistance to manganese, the medium was supplemented with 5 mM MnSO_4_ [42], which provides notable but not complete growth inhibition of the CRN strain. In the control YPD medium, Mn^2+^ concentration was 0.004 mM. Growth curves were obtained by measuring the optical density (OD) at 530 nm in a 0.3 cm cuvette, 3 replicates per experiment. The cell concentration was measured in a hemocytometer, 10 samples for each experiment. For the analyses of polyphosphates and manganese content, the cells were harvested at 5000 g for 20 min, washed twice with sterile distilled water. The samples for RNA-Seq library construction were washed thrice.

### 2.2. RNA Extraction and Sequencing for Transcriptome Profiling

The CRN and CRN/PPN1 strains were grown in control YPD and YPD with 5 mM of MnSO_4_. After cultivation for 12 h (control, both strains), 40 h (manganese, CRN) and 16 h (manganese, CRN/PPN1), the cells were harvested by centrifugation at 5000× *g* for 10 min and washed three times with distilled water at 0 °C, centrifuged after each washing, and placed in RNALater. Two replicates were obtained from two simultaneous cultivations in control YPD and manganese-containing YPD. Total RNA extraction was performed using an RNeasy Plant Kit (Qiagen, Venlo, Netherlands) according to the manufacturer’s protocol—with the addition of Plant RNA Isolation aid reagent (Ambion/ThermoFisher Scientific, Waltham, MA, USA) to lysis buffer RLT. Illumina cDNA libraries were constructed using the TruSeq RNA Sample Prep Kits v2 (Illumina, San Diego, CA, USA) according to the manufacturer’s protocol. The libraries were sequenced on the Illumina NextSeq 500 (Illumina, San Diego, CA, USA). The total sequenced library size was no less than 3 million reads per replicate. The raw and processed RNA-Seq data are deposited in GEO under accession number GSE122987.

### 2.3. Processing and Analysis of RNA-Seq Data

Adapter sequences were trimmed with Trimmomatic v0.36 (ILLUMINACLIP:TruSeq3-SE.fa:2:30:10 LEADING:3 TRAILING:3 SLIDINGWINDOW:4:30 MINLEN:36). Read mapping and counting was performed with STAR v2.5.3a (default parameters) using Ensembl *S. cerevisiae* R64-1-1.90 genome annotation (genome-build-accession GCA_000146045.2, UCSC sacCer3). For all samples, no less than 88% of the reads were mapped uniquely. The resulting gene lists were filtered to contain only genes reaching at least 2 counts per million in at least four of the eight libraries. Read counts were normalized using the edgeR [43] TMM approach. A principal component analysis of the resultant normalized and log-transformed matrices was done in the R environment. Differential expression was assessed with edgeR using a generalized linear model, accounting for the interaction between the strain (CRN, CRN/PPN1) and growth conditions (control or manganese excess medium). The genes with the FDR-adjusted *p*-value ≤ 0.05 and |log_2_ (Fold Change)| ≥ 1 were considered differentially expressed. The gene ontology (GO) enrichment analysis was performed with the YeastMine software [44]. The read counts and differential gene expression data are presented in Tables S3 and S4.

### 2.4. Polyphosphate and Polyphosphatase Activity Assay

For polyphosphate assay, the cells were cultivated for 24 h (CRN, CRN/PPN1 and CRN/PPX1 strains, control growth; CRN/PPN1 strain, growth in the presence of 5 mM MnSO_4_) and for 50 h (CRN and CRN/PPX1 strains, growth in the presence of 5 mM MnSO_4_).

The acid-soluble polyphosphate was extracted from biomass samples by the twofold treatment, with 0.5 M HClO_4_ at 0 °C for 15 min with continuous stirring. The cells were precipitated at 4000 g. In supernatants, the Pi and polyP amounts were assayed as described earlier [45]. The amount of acid-insoluble polyphosphate in the remaining biomass was assayed by the Pi content after twofold treatment with 0.5 M HClO_4_ at 90 °C for 20 min. The polyphosphatase activities were measured in cell-free extracts with the polyphosphate of 65 phosphate residues as described earlier [40].

### 2.5. Manganese Assay

The CRN, CRN/PPN1, and CRN/PPX1 strains were cultivated in YPD medium supplemented with 5 mM MnSO_4_. The cells were cultivated to the logarithmic growth stage: For 40 h (CRN and CRN/PPX1) and for 16 h (CRN/PPN1). The manganese content was assayed by atomic absorption spectroscopy after biomass sample burning at 150 °C in 32% HClO_4_.

### 2.6. The Assay of H_2_O_2_ MICs

The minimum inhibitory concentrations (MICs) of H_2_O_2_ were determined by inoculation of cell samples (standardized by culture density) in sterile plates with covers in YPD medium supplemented with H_2_O_2_. After 24-h cultivation, culture densities were measured using Sapphir plate photometers (Moscow, Russia) at 600 nm.

### 2.7. Fluorescence and Light Microscopy

Live and dead cells were stained using a LIVE/DEAD Fungal Light Yeast Viability Kit (Molecular Probes Inc. / ThermoFisher Scientific, Waltham, MA, USA) according to the manufacturer’s instructions. Yeast cultures were stained without washing and incubated with the staining reagent for 15 min at 37 °C. In case of growth inhibition in the presence of manganese, the cell culture was concentrated by centrifugation before staining. The samples were examined using a fluorescent microscope AXIO Imager A1 ZEISS (Oberkochen, Germany) with a filter kit 56 (ZEISS) at a wavelength of 450–500 nm (excitation) and 600–650 nm (emission). The number of stained cells was calculated on 3 to 5 micrographs.

1,2,3-dihydrorhodamine was used for ROS staining [46]. Cell culture samples (1 mL) were centrifuged at 5000× *g* for 5 min, washed twice with MilliQ water and suspended in 1 mL of MilliQ water. After the addition of 0.02 mL 1,2,3-dihydrorhodamine (Sigma-Aldrich, St. Louis, MO, USA) (stock solution 0.5 mg/mL in DMSO), the cells were incubated at 30 °C and 1000 rpm in a Thermomixer (Biosan, Riga, Latvia). Then the samples were centrifuged and washed twice with MilliQ water. The Nikon Eclipse FN 1 microscope with Intenselight C-HGFI HG Precentered Fiber Illuminator (Nikon, Tokyo, Japan) was used to make micrographs. The emission was registered with the bandpass filter 420–620 nm.

## 3. Results

### 3.1. CRN/PPN1 Strain Exhibits a Decreased PolyP Level and Enhanced Resistance to Manganese

**Figure 1:**
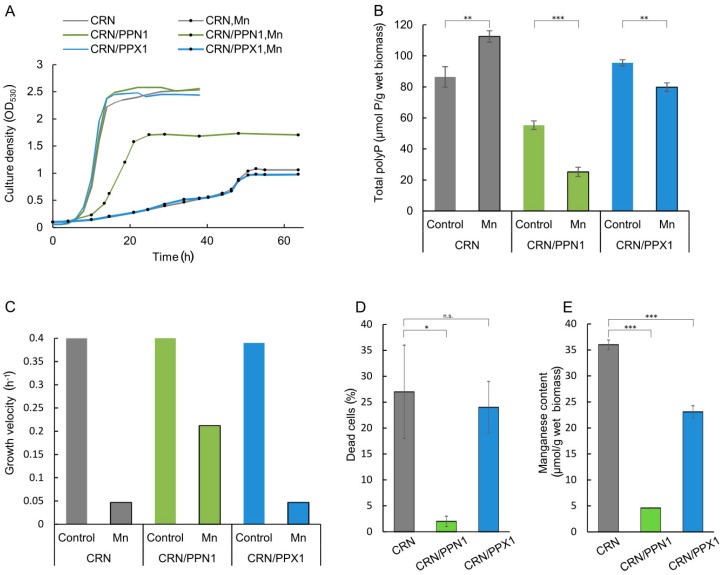
Growth characteristics and polyphosphate content in the cells of CRN, CRN/PPN1, and CRN/PPX1 strains of *S. cerevisiae* cultivated in control YPD medium with 14 mM KH_2_PO_4_ and in the same medium supplemented with 5 mM MnSO_4_. (**A**) growth curves; control medium—lines without dots; manganese excess medium—lines with dots; CRN is shown in gray, CRN/PPN1 in green, and CRN/PPX1 in blue; (**B**) growth velocities, logarithmic growth stage; (**C**) manganese content in the cells grown in the presence of 5 mM MnSO_4_, logarithmic growth stage: CRN (40 h growth), CRN/PPN1 (16 h growth), and CRN/PPX1 (40 h growth); (**D**) the percentage of dead cells in the logarithmic growth stage in the presence of 5 mM MnSO_4_; (**E**) total polyphosphate content in control and manganese grown cells, stationary growth stage. Three replicates were performed for each experiment; mean ± s.d. is shown on panels (**B**,**D**,**E**). Two-sample t-test was used to assess the statistical significance: *** *p*-value < 0.001, ** *p*-value < 0.01, * *p*-value < 0.05, n.s.—not significant.

The parent CRN strain (Δ*ppn1* mutant), the CRN/PPN1 strain (overproducing Ppn1 polyphosphatase), and the CRN/PPX1 strain (transformed by the same vector but carrying the *PPX1* gene encoding Ppx1 polyphosphatase) showed no difference in the control growth in YPD medium (Figure 1A). The polyphosphatase activity in the cell-free extracts was higher in the respective overproducing strains (Table S1). The polyP level in CRN/PPN1 cells was lower as compared to CRN and CRN/PPX1 (Figure 1B). A decrease in the polyP level was more pronounced in the acid-soluble polyP fraction (Table S2), as it was observed in selective YNB medium [40]. In the presence of excess manganese, the polyP level was increased in CRN cells, remained almost unchanged in CRN/PPX1 cells, and showed a further decrease in CRN/PPN1 cells (Figure 1B).

The manganese excess inhibited the growth of CRN and CRN/PPX1 strains (Figure 1A,C) similar to other *S. cerevisiae* strains [42]. Surprisingly, the growth inhibition in the medium with manganese excess was dramatically lower for polyP-deficient CRN/PPN1. Furthermore, manganese excess induced a notable cell death of the CRN and CRN/PPX1 strains (Figure 1D and Figure S1), while CRN/PPN1 was more resistant and accumulated a lower amount of manganese (Figure 1E).

### 3.2. Transcriptome Profiling Reveals the Pre-Adapted State of the CRN/PPN1 Strain

To explore the mechanism of CRN/PPN1 resistance to manganese, we performed transcriptome profiling (RNA-Seq) for the CRN and CRN/PPN1 strains in the control and in manganese excess media.

**Figure 2:**
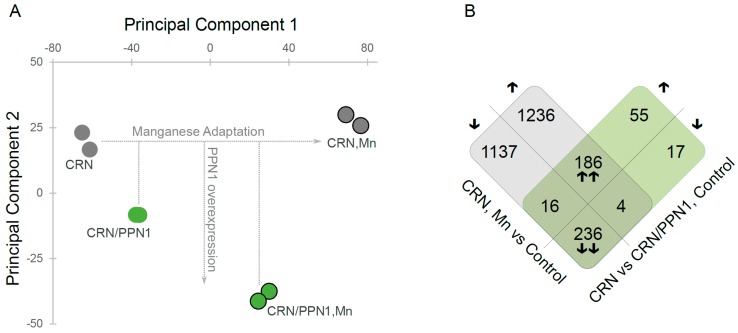
Gene expression profile of the CRN/PPN1 strain is similar to that of the manganese-adapted CRN strain. CRN and CRN/PPN1 were grown in control YPD with 14 mM KH_2_PO_4_ and in the same medium supplemented with 5 mM MnSO_4_. (**A**) Principal component analysis of gene expression data: (green circles) CRN/PPN1 samples; (gray circles) CRN samples. Circles denoting the samples grown in manganese excess conditions (Mn) are outlined. (**B**) Venn-like diagram for genes up- and down-regulated in (gray) CRN in control YPD versus CRN in manganese-supplemented YPD; (green) CRN/PPN1 as compared to CRN, both in control YPD. Each cell shows the number of genes; the direction of expression change (up- or down-regulation) is shown with arrows. Up- or down-regulated genes in both comparisons are indicated by double arrows.

To investigate global gene expression changes in CRN and CRN/PPN1 strains, we used a principal component analysis (Figure 2A) of the quantitative gene expression data (Table S3). The first and the second principal components (PCs) account for 70% of variance and clearly segregate the samples: PC1 reflects the expression changes arising from the adaptation to manganese, while PC2 reflects the changes resulting from Ppn1 overexpression. Interestingly, CRN/PPN1 samples were shifted towards the manganese adapted state even in the control medium. This observation is also supported by a comparison of gene sets up- and down-regulated in CRN/PPN1 and upon manganese adaptation (Figure 2B), which are strikingly similar, with only a few genes uniquely differentially expressed in CRN/PPN1.

To investigate this phenomenon, we assessed differential gene expression (Table S4) for the samples grown in manganese versus control medium, and used the gene ontology enrichment analysis.

The adaptation of the parent CRN strain to manganese follows the same pattern as we have observed earlier for the CRY strain [47]. The up-regulated genes were enriched with multiple GO terms reflecting stress response, cell wall, and membrane organization (multiple GO terms, *p* < 10^−7^). The down-regulated genes were enriched with all types of protein biosynthesis and ribosome-related GO terms, which clearly agrees with growth suppression under manganese excess (Figure 1A,C).

The CRN/PPN1 adaptation follows the same pattern as that of the CRN strain, with a few additions of GO-terms related to phosphate metabolic processes (GO:0006796 and GO:0006793, *p* < 10^−4^), which is associated with changes in the polyP content (Figure 1B).

Additionally, we estimated differential gene expression in the control medium for CRN/PPN1 versus CRN samples. This analysis confirmed the pre-adapted state of the CRN/PPN1 strain, with up-regulation of the genes responsible for cellular response to external stimulus (GO:0071496 and GO:0009991, *p* < 10^−5^), plasma membrane components (GO:0005886, *p* < 10^−4^), and, importantly, oxidation-reduction process (GO:0055114, *p* < 10^−5^). Of note, in manganese-adapted CRN/PPN1 samples, slight enrichment of the genes responsible for oxidoreductase activity (GO:0016491, *p* ~0.01) was also detected.

Thus, Ppn1 overexpression leads to general adaptive processes and, specifically, oxidation-reduction pathways. This particular feature facilitates CRN/PPN1 adaptation to manganese excess.

### 3.3. CRN/PPN1 Strain Has Increased ROS Level and H_2_O_2_ Resistance

**Figure 3:**
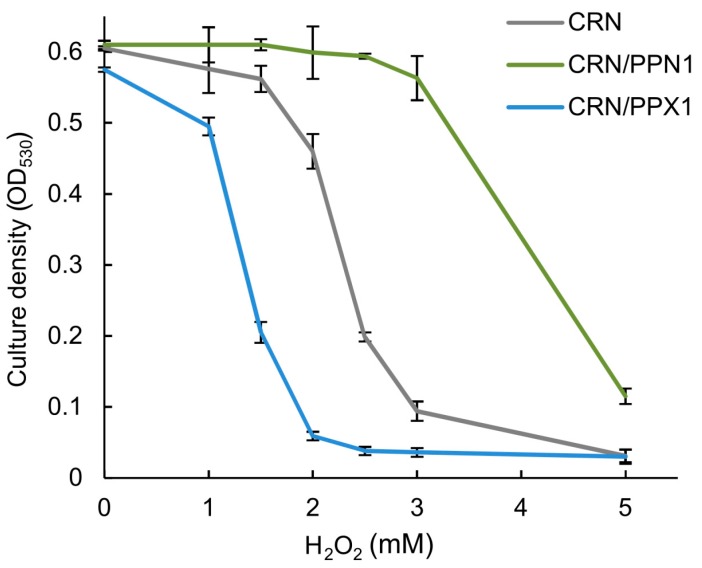
The effect of H_2_O_2_ on the growth of the CRN, CRN/PPN1, and CRN/PPX1 strains of *S. cerevisiae* in YPD medium. CRN is shown in gray, CRN/PPN1 in green, and CRN/PPX1 in blue. Three replicates were performed for each experiment; mean ± s.d. is given.

**Figure 4:**
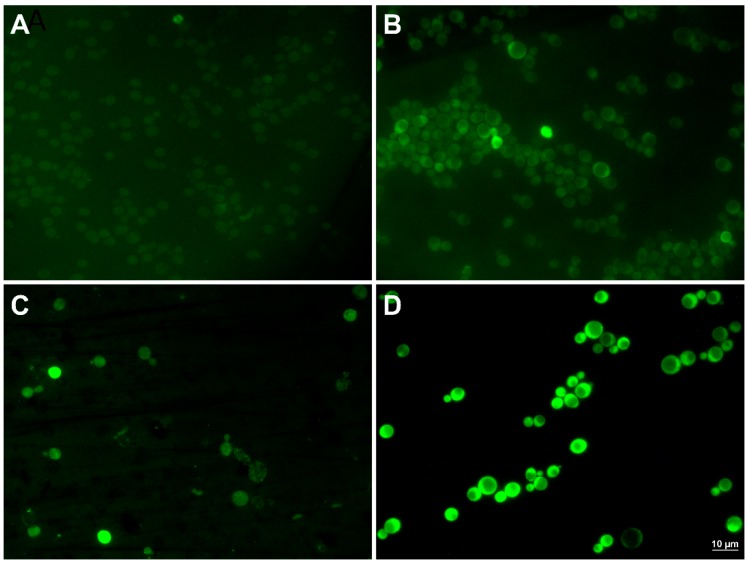
The micrographs of the cells of CRN and CRN/PPN1 strains of *S. cerevisiae* stained with 1,2,3-dihydrorhodamine. The strains were cultivated in control YPD medium with 14 mM KH_2_PO_4_, and in the same medium supplemented with 5 mM MnSO_4_ to the logarithmic stage (control—12 h growth, both strains; 5 mM manganese—40 h growth, CRN; 16 h growth, CRN/PPN1). (**A**) CRN, control YPD; (**B**) CRN/PPN1, control YPD; (**C**) CRN, manganese excess; (**D**) CRN/PPN1, manganese excess.

The global transcriptome analysis suggested that the CRN/PPN1 strain has a pre-adapted state realized through up-regulation of the oxidation-reduction pathways. We estimated MICs of H_2_O_2_ for CRN, CRN/PPX1, and CRN/PPN1 growth. Indeed, the CRN/PPN1 strain showed enhanced H_2_O_2_ resistance as compared to CRN or CRN/PPX1. The average MICs of H_2_O_2_ were 2.5, 1.5, and 4.0 mM for CRN, CRN/PPX1, and CRN/PPN1, respectively (Figure 3). Additionally, we compared the reactive oxygen species (ROS) levels in CRN and CRN/PPN1—both in the control and manganese excess media. The staining with 1,2,3-dihydrorhodamine showed a higher ROS level in CRN/PPN1 cells in both cases (Figure 4, Supplementary Figure S2).

### 3.4. An Increased Manganese Resistance of CRN/PPN1 Is Realized through PHO-Pathway Genes

To understand the enhanced manganese resistance of the CRN/PPN1 strain, we paid special attention to particular groups of genes (Table 1).

First of all, with manganese excess, the genes encoding manganese transport of the plasma membrane, ER and Golgi (*SMF1*, *SMF2*, *PMR1*), are up-regulated, while the genes of vacuolar transporters are either stable (*CCC1*) or down-regulated (*GDT1P*). In the control medium, there are no major changes in expression of the above genes in CRN/PPN1 versus CRN. The expression of the putative manganese chelator encoded by *MNC1* [47] is specific to manganese excess.

The plasma membrane ATPase Pma2 is essential for manganese adaptation [47]. It is up-regulated in manganese-grown cells of both strains and in CRN/PPN1 grown in the control medium, although with a weak significance (FDR ~0.08). 

Among the genes of polyP hydrolysis enzymes, we should mention *DDP1*, which is down-regulated in the presence of manganese excess, which accounts for the increase in polyP content in CRN cells. All subunits of the polyP synthesis VTC complex are down-regulated in all cases.

Regarding the known phosphate transporters, the genes encoding Pho89 and Pho84 are known to be regulated by the transcription factor Pho4. However, in our case, the changes in Pho89 and Pho84 expression correlate neither with Pho4 differential expression, nor with each other. It is noteworthy that *PHO84* is down-regulated both under manganese excess and in CRN/PPN1 in the control medium, which is in agreement with decreased manganese uptake in CRN/PPN1 cells. 

Additionally, we have found two unannotated genes, *PHM6* and *PHM7*, that were supposed to be regulated by phosphate levels [48]. Upon manganese excess, they are strongly differentially expressed in the opposite directions: *PHM6* is down-regulated, while *PHM7* is up-regulated, displaying the highest reachable fold change among the genes—up to 5% FDR-corrected *p*-value in the comparisons CRN/PPN1 versus CRN (control) and CRN (control versus manganese excess). Strong up-regulation of *PHM7* suggests its essential role in manganese adaptation, and suggests studying the survival of the Δ*phm7*-null mutant under manganese excess.

### 3.5. Δphm7 Has Reduced Resistance to Manganese

We have compared the growth characteristics of the Δ*phm7*-null mutant under the control and manganese excess conditions to confirm the role of Phm7 in resistance to manganese. The Δ*vmr1* strain was used as a positive control. *VMR1* encodes the vacuolar membrane protein that is an ABC transporter of the MRP subfamily. The deletion of this gene enhanced the sensitivity to cadmium [49] and lead [50], and, according to our data, *VMR1* was significantly up-regulated in CRN/PPN1 (as compared to CRN) and in manganese excess conditions.

**Figure 5:**
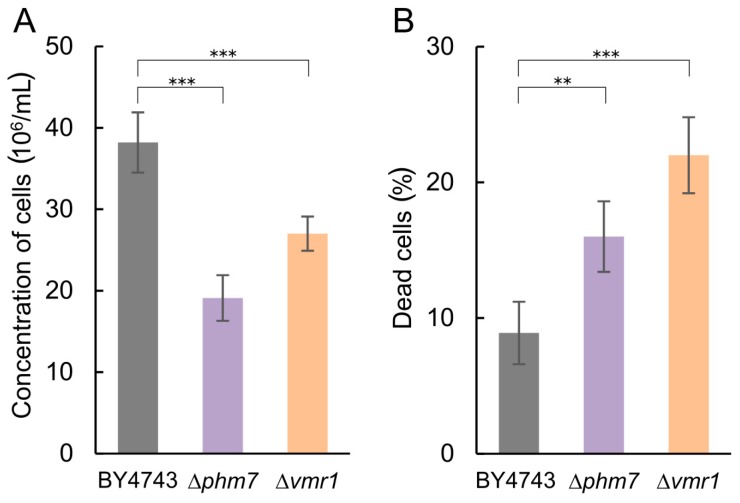
Growth characteristics of the BY4743 parent strain and Δ*phm7* and Δ*vmr1* knockout strains of *S. cerevisiae* cultivated in YPD medium with 14 mM KH_2_PO_4_ and 5 mM MnSO_4_ to the stationary growth stage (72 h). (**A**) cell concentration, (**B**) percentage of dead cells. Three replicates were performed for each experiment; mean ± s.d. is shown. Two-sample t-test was used to assess the statistical significance: *** *p*-value < 0.001, ** *p*-value < 0.01.

**Figure 6:**
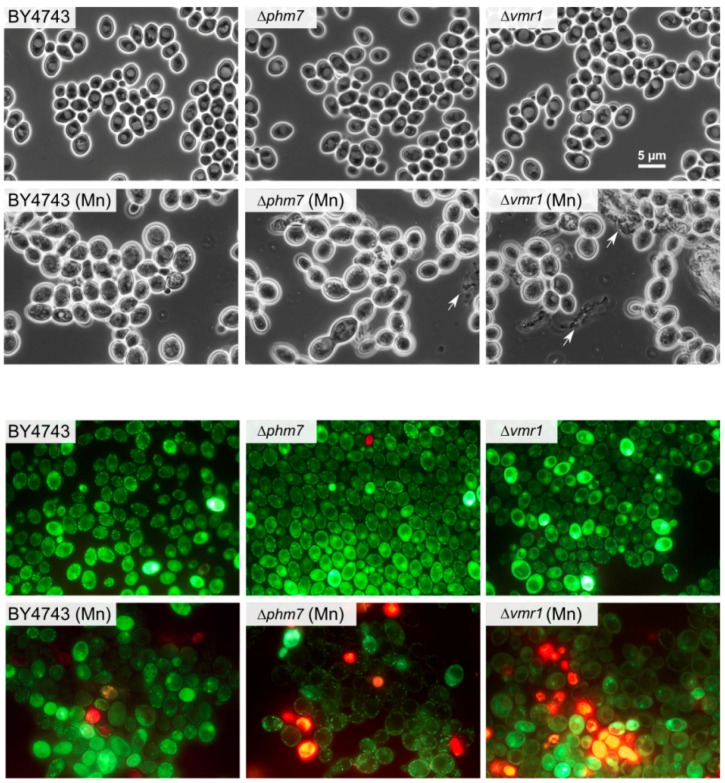
The micrographs of the cells of the parent BY4743 strain and Δ*phm7* and Δ*vmr1* knockout strains of *S. cerevisiae* cultivated in YPD medium with (Mn) and without 5 mM MnSO_4_ to the stationary growth stage (72 h). Top—phase contrast, bottom—LIVE/DEAD Fungal Light Yeast Viability Kit staining.

The tested strains grew similarly in the YPD medium; in the presence of 5 mM manganese, the growth of Δ*phm7* and Δ*vmr1* was inhibited more than that of the parent BY4743 strain, the cell concentrations of the mutants were lower, and the number of dead cells was significantly higher than in the parent strain (Figure 5). The light microscopy of the mutant cultures revealed large-scale cell lysis in the stationary phase (Figure 6).

## 4. Discussion

PolyP is widely recognized as an anionic polymer that binds and de-toxifies heavy metal ions [41]. However, in yeast, particular mechanisms of polyP-involving detoxification are understudied. Manganese-adapted yeast cells contain longer polyP chains [42], but this could be a side effect of VTC4 activation by manganese ions [28]. PolyP content increases during the elongated lag phase in manganese excess conditions and decreases at the active growth stage [42]. It is known that the excess of manganese ions results in oxidative stress, while manganese-phosphate complexes provide antioxidant activity [26]. Thus, phosphate released through polyP degradation in yeast cells could allow for manganese adaptation.

In bacteria, polyP is known to be necessary for oxidative stress adaptation [2] due to its ability to act as a chaperone [24,25]. In our study, we found evidence for polyP involvement in stress response in yeast: The reduced polyP content through Ppn1 polyphosphatase overexpression leads to a significant alteration in expression of genes responsible for manganese and oxidative stress adaptation. Manganese homeostasis in *S. cerevisiae* involves numerous proteins [26,51,52,53]. The adaptation of yeast cells to toxic concentrations of manganese ions involves—in particular, the transport systems responsible for manganese compartmentalization, the systems of phosphorus transport and polyphosphate accumulation, as well as other membrane proteins. In this study, we identified new proteins contributing to manganese adaptation in *S. cerevisiae*. The protein encoded by *PHM7* is essential for this process. The Phm7 protein sequence contains several transmembrane domains [54], and the protein is localized in the plasma membrane [55]. Its ortholog CaPhm7 in *Candida albicans* is also localized in the plasma membrane, and participates in drug resistance and filamentous growth [56,57]. The cells lacking *CaPHM7* are sensitive to SDS and ketoconazole, but resistant to rapamycin and zinc [56]. This evidence suggests that Phm7 is a membrane transporter of divalent metal cations.

Another important observation is the role of Pho84 in oxidative stress response, in addition to its known role in phosphate and manganese transport. Pho84 is the major high-affinity plasma membrane phosphate importer of *S. cerevisiae* and an important element of phosphate homeostasis [58]. Pho84 is a phosphate transceptor: It transports phosphate and mediates rapid phosphate activation of the protein kinase A (PKA) pathway [59]. This protein also acts as a divalent cation transporter [52]. The knockout strain shows multiple phenotypic changes: Poor growth on a low-phosphate medium, constitutive up-regulation of the PHO pathway, and negligible levels of polyP, enhanced arsenate [60], and manganese resistance [61]. The overexpression of *PHO84* induces enhanced heavy metal accumulation [62].

In our case, *PHO84* is down-regulated in the CRN/PPN1 strain that exhibits manganese and resistance to peroxide. In *C. albicans*, Pho84 participates in oxidative stress response: The Δ*pho84* mutant accumulates intracellular ROS in the absence of extrinsic oxidative stress and hyperactivates oxidative stress signaling [63]. Thus, *PHO84* down-regulation enhances resistance to manganese by decreasing metal ion uptake and mobilizing oxidative stress signaling. This down-regulation can also cause resistance to peroxide.

The mechanisms of *PHO84* down-regulation in CRN/PPN1 are still in question. The intracellular phosphate level may be increased as a result of polyP hydrolysis in the PPN1 overproducing strain CRN/PPN1, which leads both to *PHO84* down-regulation and an increase of the ROS level. A decrease of the polyP level reduces the antioxidant potential of the cell, because polyP plays an antioxidant role [24], additionally increasing the ROS level. As a result, the cells mobilize their stress response pathways, thereby providing, together with *PHO84* down-regulation, the manganese and peroxide resistance of the CRN/PPN1 strain.

The reduced polyP of CRN/PPN1 was expected to lead to decreased resistance to manganese. Instead, we found the opposite—i.e., the increased resistance achieved through the pre-adaptive state. PolyP deficiency does not affect the cell phenotype and growth in normal conditions, but acts as a cellular signal leading to major changes in the gene expression pattern that facilitates stress adaptation.

## Figures and Tables

**Table 1:** Differential expression of the selected genes responsible for manganese transport and belonging to the PHO pathway. The cases of differential expression with FDR-corrected *p*-value ≤ 0.05 are marked in bold.

Gene ID	Gene Name	Gene Function	Log_2_ Fold Change		
			CRN, Mn^2+^ vs. Control	CRN/PPN1, Mn^2+^ vs. Control	CRN/PPN1 vs. CRN, Control
**Manganese Transporters and Chelators**					
YOL122C	*SMF1*	Cell surface manganese transporter	**0.96**	0.05	0.14
YHR050W	*SMF2*	ER membrane manganese transporter	**1.34**	**0.82**	0.30
YGL167C	*PMR1*	High affinity Ca^2+^/Mn^2+^ P-type ATPase of Golgi	**1.27**	**0.82**	0.08
YLR220W	*CCC1*	Vacuolar Fe^2+^/Mn^2+^ transporter	−0.09	0.00	0.56
YBR187W	*GDT1P*	Calcium and manganese transporter of Golgi	**−1.62**	**−0.78**	**−0.87**
YBR056W-A	*MNC1*	Putative manganese chelating CYSTM-family protein	**2.32**	**1.24**	−0.02
**Plasma Membrane ATPases**					
YGL008C	*PMA1*	Plasma membrane H^+^-ATPase	**0.51**	**0.98**	−0.03
YPL036W	*PMA2*	Plasma membrane H^+^-ATPase	**5.85**	**5.90**	3.34
**Polyphosphate Hydrolyzing Enzymes**					
YDR452W	*PPN1*	Exo/endopolyphosphatase	−0.27	**10.67**	**9.30**
YHR201C	*PPX1*	Exopolyphosphatase	−0.06	0.14	−0.12
YNL217W	*PPN2*	Endopolyphoshatase	0.00	**−0.58**	**−0.63**
YOR163W	*DDP1*	Diadenosine and diphosphoinositol endopolyphosphatase	**−1.00**	**−0.96**	−0.46
**Subunits of the VTC Complex Involved in Polyphosphate Biosynthesis**					
YER072W	*VTC1*	Regulatory subunit involved in membrane trafficking and vacuolar polyphosphate accumulation	**−5.48**	**−5.05**	**−2.94**
YFL004W	*VTC2*		**−1.59**	**−1.52**	**−0.98**
YPL019C	*VTC3*		**−1.18**	**−2.51**	**−2.33**
YDR089W	*VTC5*		**−0.78**	**−0.96**	0.07
YJL012C	*VTC4*	Vacuolar membrane polyphosphate polymerase	**−0.97**	**−1.48**	**−1.90**
**Phosphate Transport**					
YFR034C	*PHO4*	Activates transcription in response to phosphate limitation; function is regulated by phosphate availability	0.82	0.81	−0.52
YML123C	*PHO84*	High-affinity inorganic phosphate transporter; also low-affinity manganese transporter, regulated by Pho4p	**−2.29**	**−6.35**	**−5.59**
YBR296C	*PHO89*	Plasma membrane Na^+^/Pi cotransporter, transcription regulated by Pho4p	**3.52**	0.18	**−4.37**
YCR037C	*PHO87*	Low-affinity inorganic phosphate transporter	0.14	0.36	−0.26
YJL198W	*PHO90*	Low-affinity phosphate transporter	0.40	**0.99**	−0.02
YNR013C	*PHO91*	Low-affinity vacuolar phosphate transporter	0.39	**0.73**	0.00
YDR281C	*PHM6*	Putative transport protein	**−6.0**	**−5.97**	**−3.52**
YOL084W	*PHM7*	Putative transport protein	**12.01**	**11.47**	**8.22**

## Supplementary Materials

The following are available online at https://www.mdpi.com/2073-4409/8/5/461/s1, Figure S1: The micrographs of *S. cerevisiae* cells cultivated in YPD medium with 5 mM MnSO_4_ for logarithmic growth stage; Figure S2: The micrographs of the cells of CRN and CRN/PPN1 strains of *S. cerevisiae* stained with 1,2,3-dihydrorhodamine; Table S1: The *S. cerevisiae* strains used in the study; Table S2: The Pi and polyP content in *S. cerevisiae* cells grown in control YPD medium and in the presence of 5mM MnSO_4_; Table S3: Gene counts estimated from the RNA-Seq data; Table S4: Differential gene expression data.
