# Supplementary material for: The Reduced Level of Inorganic Polyphosphate Mobilizes Antioxidant and Manganese-Resistance Systems in Saccharomyces cerevisiae
Source: Cells. 2019 May 15;8(5):461. doi: 10.3390/cells8050461 (PMC6562782; doi:10.3390/cells8050461)
Supplement: Supplementary file 1 [file cells-08-00461-s001.zip › cells-496981-2nd revise-supplementary/Figure S1.pdf]

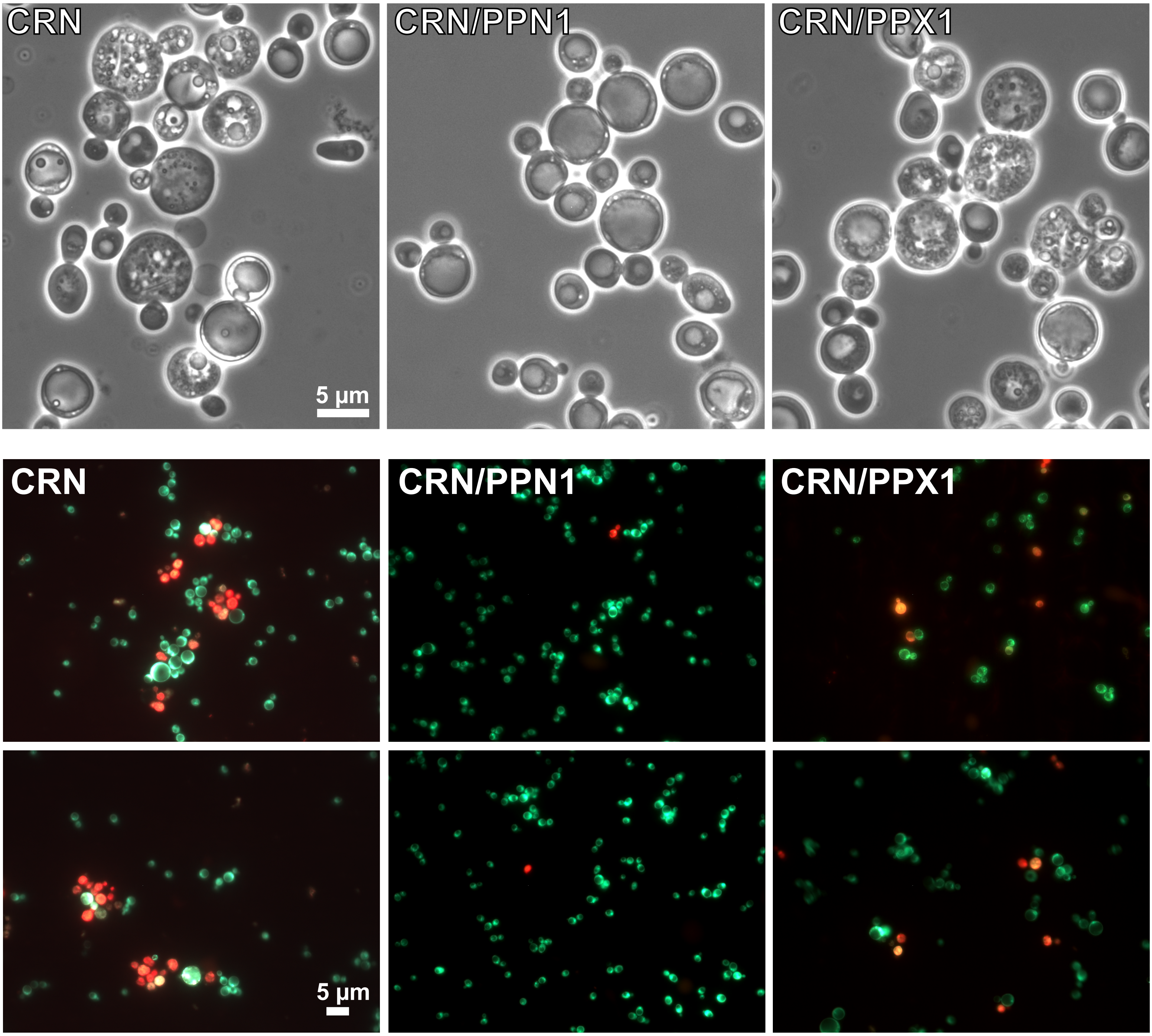

Figure S1. The micrographs of *S. cerevisiae* cells cultivated in YPD medium with 5 mM $MnSO_4$ for logarithmic growth stage: CRN (40 h of growth), CRN/PPN1 (16 h of growth), and CRN/PPX1 (40 h of growth). Top row – phase contrast, bottom rows – staining with LIVE/DEAD Fungal Light Yeast Viability Kit. Strain names are given in the top row images.
