# Supplementary material for: The Reduced Level of Inorganic Polyphosphate Mobilizes Antioxidant and Manganese-Resistance Systems in Saccharomyces cerevisiae
Source: Cells. 2019 May 15;8(5):461. doi: 10.3390/cells8050461 (PMC6562782; doi:10.3390/cells8050461)
Supplement: Supplementary file 1 [file cells-08-00461-s001.zip › cells-496981-2nd revise-supplementary/Figure S2.pdf]

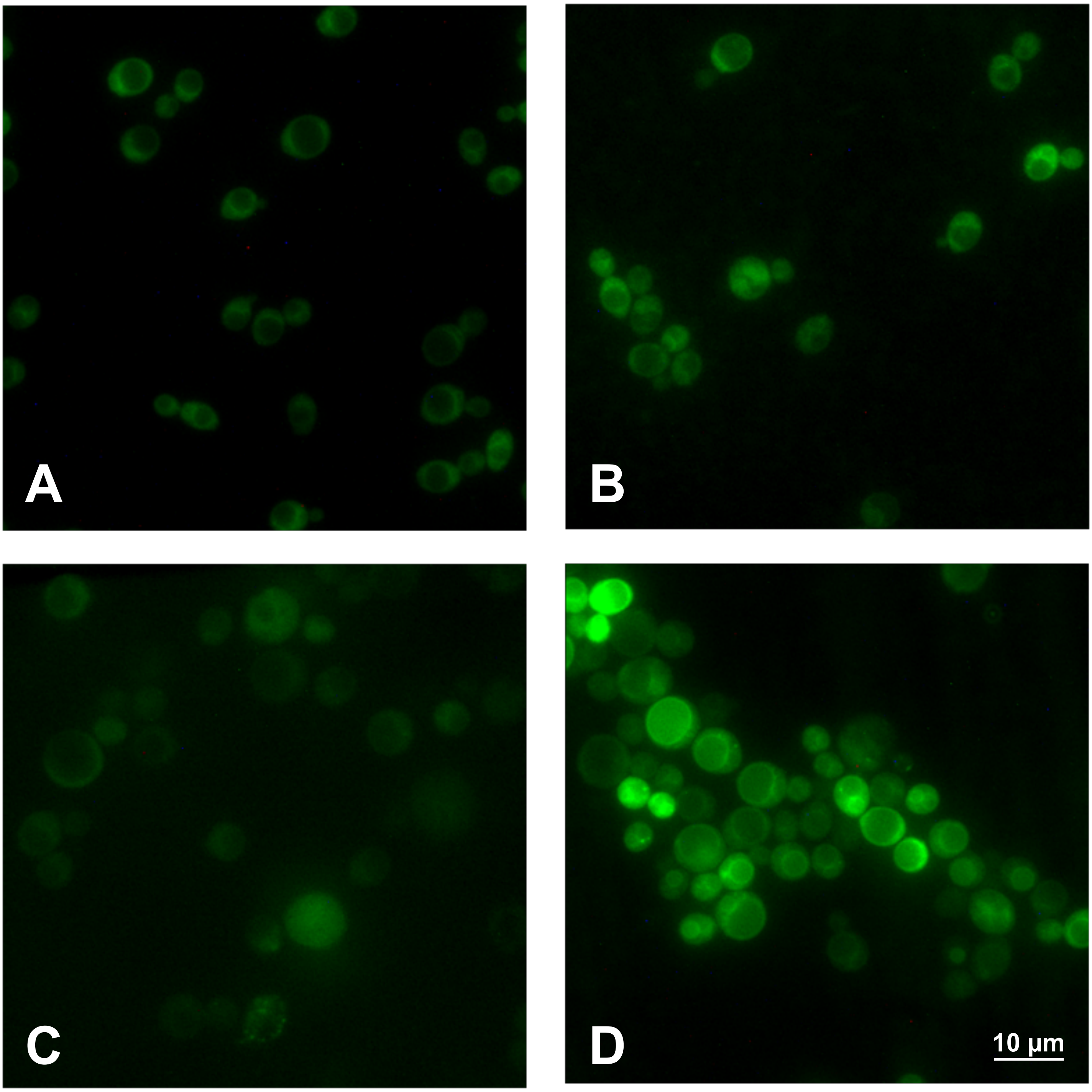

Figure S2. The micrographs of the cells of CRN and CRN/PPN1 strains of *Saccharomyces cerevisiae* stained with 1,2,3-dihydrorhodamine. The strains were cultivated in control YPD medium with 14 mM KH_2PO_4 and in the same medium supplemented with 5 mM $MnSO_4$ to the logarithmic stage (control - 12 h growth, both strains; 5 mM manganese – 40 h growth, CRN; 16 h growth, CRN/PPN1). (A) – CRN, control YPD; (B) – CRN/PPN1, control YPD; (C) – CRN, manganese excess; (D) – CRN/PPN1, manganese excess.
