# Supplementary material for: The Reduced Level of Inorganic Polyphosphate Mobilizes Antioxidant and Manganese-Resistance Systems in Saccharomyces cerevisiae
Source: Cells. 2019 May 15;8(5):461. doi: 10.3390/cells8050461 (PMC6562782; doi:10.3390/cells8050461)
Supplement: Supplementary file 1 [file cells-08-00461-s001.zip › cells-496981-2nd revise-supplementary/Table S1,S2.pdf]

Table S1. The *S. cerevisiae* strains used in the study.

Strain	Genotype	Polyphosphatase-related features	Polyphosphatase activity in cell-free extract, U/mg protein (logarithmic phase)	References
CRN	*MAT*a *ade2 his3 ura3 ppn1Δ::CgTRP1*	PPN1 polyphosphatase null mutant	0.54	Sethuraman A, Rao NN, Kornberg A. The endopolyphosphatase gene: essential in *Saccharomyces cerevisiae,* Proc Natl Acad Sci USA 2001; 98:8542–7.
CRN/PPN1	*MAT*a *ade2 his3 ura3 ppn1Δ::CgTRP1* transformed with vector pMB1/PPN1 Sc	PPN1 polyphosphatase overexpression	4.73	Eldarov MA, Baranov MV, Dumina, MV, Shgun AA, et al. (2013) Polyphosphates and exopolyphosphatase activities in the yeast *Saccharomyces cerevisiae* under overexpression of homologous and heterologous *PPN1* genes. Biochemistry (Moscow) 2013;78: 946–53.
CRN/PPX1	*MAT*a *ade2 his3 ura3 ppn1Δ::CgTRP1* transformed with vector pMB1/PPX1 Sc	PPX1 polyphosphatase overexpression	14.5	Lichko LP, Eldarov MA, Dumina MV, Kulakovskaya TV. PPX1 gene overexpression has no influence on polyphosphates in *Saccharomyces cerevisiae.* Biochemistry (Moscow) 2014;79: 1211–5.

Table S2. The Pi and polyP content in *S. cerevisiae* cells grown in control YPD medium (24 h) and in the presence of 5mM $MnSO_4$ (24 h of growth for CRN/PPN1 strain and 50 h of growth for CRN and CRN/PPX1 strains).

Strain	Growth conditions	Mineral phosphorus compounds, µmol P/wet biomass		
		P_i	Acid soluble polyP	Acid insoluble polyP
CRN	Control	37.9±1.1	52.0±4.8	34.4±1.9
	+Mn^{2+}	18.6±0.7	64.0±2.5	48.5±1.9
CRN/PPN1	Control	40.8±0.9	24.6±0.5	30.7±2.3
	+Mn^{2+}	38.0±4.0	7.8±2.8	17.4±0.2
CRN/PPX1	Control	38.0±0.7	57.3±1.0	38.2±1.0
	+Mn^{2+}	33.5±5.8	44.5±1.5	35.3±1.9
